# Relationship between Fixed Dental Crowns and Volatile Sulphur Compounds

**DOI:** 10.3390/ijerph18031283

**Published:** 2021-02-01

**Authors:** Hamad Alzoman, Syed Rashid Habib, Sultan Alghamdi, Hebah Al-Juhani, Rawan Daabash, Wijdan Al-Khalid, Mansour Al-Askar, Sulieman Al-Johany

**Affiliations:** 1 Department of Periodontics and Community Dentistry, College of Dentistry, King Saud University, Riyadh 11545, Saudi Arabia; malaskar@ksu.edu.sa; 2 Department of Prosthetic Dental Sciences, College of Dentistry, King Saud University, Riyadh 11545, Saudi Arabia; syhabib@ksu.edu.sa (S.R.H.); saljohany@ksu.edu.sa (S.A.-J.); 3 Internship Program, College of Dentistry, King Saud University, Riyadh 11545, Saudi Arabia; 427101153@student.ksu.edu.sa (S.A.); 428203764@student.ksu.edu.sa (H.A.-J.); 428203304@student.ksu.edu.sa (R.D.); 428203468@student.ksu.edu.sa (W.A.-K.)

**Keywords:** halitosis, oral malodor, dental prosthesis, volatile sulphur compounds

## Abstract

Objectives: The aim of this study was to investigate and compare the level of halitosis in patients with/without fixed crowns and in addition the influence of various crown parameters on halitosis was also explored. Methods: In total, 96 subjects (fixed crowns = 52; no crowns = 44) participated in the study. The levels volatile sulphur compounds (VSCs) of hydrogen sulphide (H2S), methyl mercaptan (CH3SH), and dimethyl sulphide (CH3SCH3) were evaluated with breath samples using gas chromatography and used for classification as presence or absence of halitosis. The periodontal clinical parameters for all the participants as well as the crown parameters for participants with fixed crowns were also evaluated. Cross tabulation, Chi-square test, and one-way analysis of variance tests were used for the statistical analysis and comparisons. Results: Breath samples revealed, 50 (52.1%) participants were suffering from halitosis. Out of VSCs, the level of CH3SCH3 (62.5%) was found to be the most prevalent. Significant correlations were observed between the presence of fixed crowns and oral halitosis (*p* < 0.001). Statistically significant difference in the concentration of H2S and CH3SH (*p* < 0.001) and no significance for CH3SCH3 (*p* = 0.075) between patients with/without fixed crowns was found. The presence of halitosis was more prevalent in the subjects with crown parameters (subgingival margin, over-contoured margin, open-crown margin, over-contoured and under-contoured crowns) considered clinically defective/unacceptable (*p* < 0.05). Conclusions: Presence of fixed dental crowns significantly contributes to the oral halitosis. Dental crowns with defects significantly impair the hygienic conditions and oral microflora resulting in high prevalence of halitosis.

## 1. Introduction

The word malodor, or halitosis can be defined as any disagreeable odor in expired air, regardless of whether the odorous substances originate from oral or nonoral sources [1,2]. Oral halitosis has been a condition by which more than 50% of the population suffers from [3]. Prevalence of oral malodor could be underestimated due to the fact that individuals with oral malodor do not always notice the symptoms by themselves [4,5].

The risk of halitosis is slightly more than three times higher in people over 20 years of age compared with those aged 20 years or under [6]. The origin of oral halitosis has been divided into nonoral and oral sources. The nonoral sources have been subdivided into pathological and nonpathological oral malodors [7]. Pathological oral malodors may appear as results of some systemic diseases such as diabetes mellitus, gastrointestinal conditions, irregular bowel movement, uremia, hepatic and renal failure [8,9,10]. Nonpathological oral malodors are those resulting from nonpathologic condition such as stress, empty stomach, or specific food ingestion such as herbs, garlic, and onion [11].

Oral sources of halitosis have been associated with plaque, tongue coating, decreased salivary flow rate during sleeping, food debris, smoking [9,12]. The tongue has been allocated as the prime site for oral halitosis [9,13]. In addition, some oral diseases such as mucocutaneous lesions, Periocoronitis, apthous ulcers, candidiasis, and xerostomia were associated with oral halitosis [8,14]. Studies have reported that 80–90% of oral malodor originated from the oral cavity whereas 5–8% was from ear-nose-throat (ENT) causes [8,9,15].

The main cause of halitosis is the bacterial formation of the odorous volatile sulfur compounds (VSCs) in the oral cavity. There are three major odoriferous volatile sulfur components leading to oral malodor, which are hydrogen sulphide (H2S), methyl mercaptan (CH3SH), and dimethyl sulphide (CH3SCH3). VSCs are formed as a result of bacterial putrefaction of amino acids containing sulfur molecules such as cysteine and methionine [16]. Halitosis can be identified by several procedures which are self-assessment, organoleptic measurements, and VSCs monitoring devices [17]. VSCs have been associated with oral halitosis. Hydrogen sulfide (H2S), methyl mercaptan (CH3SH), and dimethyl sulfide (CH3SCH3) were considered as the major etiology for oral halitosis [18].

Many patients who seek fixed prosthodontic treatment are conscious about halitosis, and on the other hand other groups of patients complain of halitosis after provision of the fixed crowns. Improper construction of fixed crowns impairs the hygienic conditions in oral cavity, impedes the abilities of self-cleaning with saliva, and often serve as a factor causing halitosis. These prosthesis may act as a plaque retentive factor, which might lead to oral halitosis due to the increase in the amount of bacterial plaque accumulation [19]. Costacurta et al. reported a positive correlation between halitosis and presence of both removable and fixed prostheses. It has been found that removable dentures were associated with higher levels of VSC compared to fixed prostheses [20]. Sinjari et al. compared the level of VSC one month after placing both provisional and final crowns, they have reported high level of VSC in both groups and no statistical significant differences in the level of VSC between provisional and permanent crowns [21]. Mbodj et al. conducted a study to determine the prevalence of halitosis in subjects with dental prostheses. They reported that 35.4% of the subjects diagnosed with halitosis, 72.7% of them were having fixed partial dentures (FPD) [22]. In addition, oral malodor has been associated with the presence of other oral appliances such as fixed orthodontic appliances [23,24].

Clinical studies regarding the association of the fixed dental crowns and its various clinical parameters with halitosis are scarce. Thus, the aim of this study was to investigate and compare the level of halitosis in patients with and without full coverage fixed crowns (fixed prostheses). In addition, the study also explored the influence of the various crown parameters on the halitosis in patients with fixed crowns. We hypothesized that the existence of fixed prosthesis would be associated with presence of halitosis.

## 2. Materials and Methods

This study was approved by ethical committee at College of Dentistry research center (CDRC), King Saud University (IR 0028). In this clinical study, a total of 96 patients (50 females, 46 males) attending College of Dentistry/King Saud University Riyadh, Saudi Arabia were recruited. The sample size was determined using Cohen (1988) procedure, by assuming an effect size (f) of 0.80 and with power of 90% (1 − *β* = 0.90) and at α = 0.05; the minimal number of subjects was calculated to be 77 to establish a statistical significant difference between the two study groups. An informed consent containing details of the nature of the study was given to all participants.

The participants (18–50 years) were selected based on convenience and were without any systemic diseases or chronic illnesses. For participating patients, presence of 20 natural teeth was mandatory. Patients with presence of respiratory tract diseases, tonsillitis, presence of pathology or ulcers in the oral mucous membranes, stomach disorders, antibiotic usage in the previous 3 months, and pregnancy were excluded from the study. Detailed medical and dental history of the subjects was recorded, and the participants were divided into two groups: 52 subjects with fixed dentures (group I) and 44 subjects without fixed dentures (group II).

### 2.1. Clinical Examination and Investigations

Each patient passed through dental charting by means of clinical examination and radiographs. Dental charting included documentation of dental caries, dental restorations, fixed dental appliances, and number of missing teeth. Additionally, any defective restoration was marked for further analysis. Presence of periodontal probing depth (PPD) and bleeding on probing (BOP) was assessed using a periodontal probe (Williams probe; Hu-Friedy; Hu-Friedy, Chicago, IL, USA) with Williams markings at six points around the Ramfjord’s teeth which are (16, 21, 24, 36, 41, 44). Plaque index (Pl) was used for the determination of adherent dental plaque.

### 2.2. Assessment of Crown Parameters

To assess the relationship between oral halitosis and various crown parameters (marginal fit; location of gingival margin; crown contours) they were examined clinically using dental explorer. The clinical crown parameters for the patients were evaluated and defined by the two experienced clinicians (HAZ and SAJ). In order to avoid the interexaminer bias, all the patients with defective crown parameters were examined by the examiners according to well-defined criteria, and any possible conflict was settled with mutual agreement. The location of the crown margin was examined on four surfaces (buccal; lingual; mesial; distal) and was classified into three categories (supragingival; equigingival; subgingival). Similarly, marginal fit was explored on the same four surfaces around each crown and was classified into three categories (overhang; ledge; open margin). Finally, the crown contour was examined on the buccal and lingual surfaces as (over contour; normal contour; under contour).

### 2.3. Measurement of VSCs

VSC were detected using portable Semiconductor Gas Sensor (OralChromaTM CHM-2, Abilit, Osaka, Japan) which consists of a Teflon tube column (5 mm i.d., 300 mm length) packed with 25% oxydipropionitrile supported on Celite for the gas chromatographic analysis. The column temperature was 350 °C and ambient air was used for the carrier gas. Three VSCs (hydrogen sulphide (H2S); methyl mercaptan (CH3SH); dimethyl sulphide (CH3SCH3)) were detected by a highly sensitive semiconductor indium oxide (In2O3) gas sensor. During VSC assessment, patients were asked to refrain from eating, chewing, brushing, mouth rinsing, smoking, and the use of scented cosmetics for 3 h prior to oral malodor assessment. To obtain the breath sample, all participants were instructed to breathe through their nose while keeping their mouth closed for 60 s. Then, 1 mL of breath sample was obtained using a disposable plastic syringe. Afterwards, the collected breath sample was injected immediately into the gas censor device after removing any remnant saliva from the tip of the plastic syringe. Concentration of H2S, CH3SH, and CH3SCH3 were recorded in part per billion (PPB).

### 2.4. Statistical Analysis

Statistical analysis of the collected data was carried out using SPSS software (SPSS Inc., version 24, Chicago, IL, USA). Demographic data of the participants were recorded. Mean and standard deviations of the periodontal clinical parameters were tabulated. Presence of halitosis was classified as the ability to detect VSCs in the breath samples equal to or greater than the following cognitive threshold according to Aizawa et al. (2005) (H2S ≥ 112 PPB; CH3SH ≥ 26 PPB; CH3SCH3 ≥ 8 PPB). The percentage of specific crown parameter was computed as the number of surfaces with the parameter / total number of crown surfaces examined * 100. Cross tabulation, Chi-square test, and one-way analysis of variance tests were run for the analysis and comparison of different variables on halitosis. Association of oral malodor and presence of prosthetic dental appliances was analyzed using independent *t*-test. A *p*-value of <0.05 was used to report the statistical significance of results.

## 3. Results

In total, 96 participants managed to fulfill all parts of this clinical study. The demographic details of the participants are presented in Table 1. The mean age of the study subjects was 27.19 ± 6.3 years. Most of the participants were educated and were having moderate to good oral hygiene with history of regular dental visits. The number of participants with (54.2%) or without (45.8%) fixed prostheses was almost equal (Table 1).

Frequency and level of the different clinical and tested variables is shown in Table 2. According to the breath samples collected and analyzed, 50 (52.1%) participants were classified as suffering from halitosis. Out of the three VSCs investigated the level of CH3SCH3 (62.5%) was found to be the most prevalent source of halitosis (Table 2).

**Figure 1:**
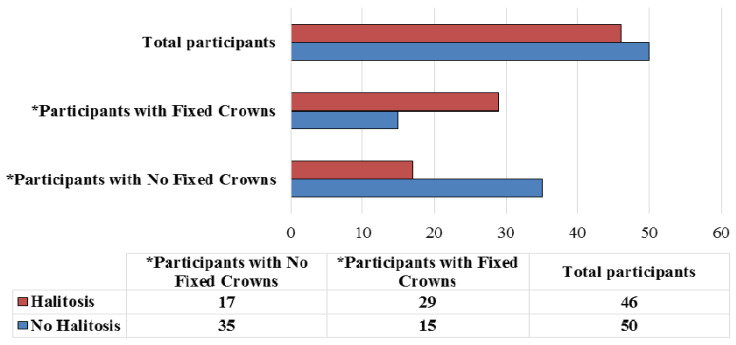
Prevalence of halitosis among the participants with or without fixed crowns (*n* = 96). *—Chi square *p*-value = 0.002.

Analysis of breath samples from 96 participants revealed detectable levels of VSCs to the level of halitosis in 46 (47.9%) of the total subjects (Figure 1). Halitosis was more prevalent among participants with fixed crowns as compared to control group. Statistically significant correlations were observed between the presence of fixed crowns and oral halitosis (*p* < 0.001) (Figure 1).

The comparison (Table 3) of mean of H2S, CH3SH, and CH3SCH3 between the two groups indicated a statistically significant difference in the concentration of H2S and CH3SH (*p* < 0.001) while no statistical significance for the concentration of CH3SCH3 (*p* = 0.075) (Table 3).

Data regarding the different crown parameters for participants with fixed crowns in relation to presence/absence of halitosis is presented in Table 4. The presence of halitosis was more prevalent in the subjects with crown parameters that are considered clinical defective/unacceptable and this was statistically significant (*p* < 0.05) (Table 4).

Analysis of clinical periodontal variables in relation to presence/absence of halitosis showed higher percentages of halitosis in participants with higher values of these variables. With increase in the values of these clinical variables the prevalence of halitosis also increased (Table 5).

## 4. Discussion

In this in-vivo research study, the prevalence of halitosis in patients with or without fixed crowns, and the influence of various crown parameters on halitosis in patients with fixed dental crowns was investigated. A substantial number of dental patients, worldwide prefer to use fixed restorations for improving their oral health related quality of life [25,26]. Ideally an optimal fixed restoration should coexist and survive in harmony with the rest of the dentition without jeopardizing the oral health status [27]. Although fixed restorations greatly impact patients’ lives, in reality the changes in the oral microbial flora due to these restorations exist and are well documented [19,20,22].

The changes in the oral flora also influences and aggravates the intraoral halitosis [27,28]. Bad breath or halitosis is a concern for millions of people and is unquestionably one of the biggest taboos around the world [4]. The breath of humans is a gas mixture of VSCs, indole, skatole, and organic acids (acetic acid, butyric acid). However, VSCs have a very high odor potential, proving that these VSCs are the major contributors to intraoral halitosis [18]. Velde et al. measured the concentration of these gases and found that the aromatic amines indole and skatole and of butyric acid were below the objectionability threshold. Their conclusion included the VSCs to be the main source of intraoral halitosis and that the roles of other compounds, like amines and organic acids, was insignificant [29].

In the current study, breath samples were taken from 96 subjects for detection of the level of VSCs using gas chromatography. Gas chromatography using a specific sulfur detector is the most reliable method for the detection of intraoral and extraoral halitosis and is considered as the gold standard for the diagnosis of halitosis [30]. It is specific and sensitive for all three volatile sulfur compounds (VSCs) i.e., hydrogen sulfide (H2S), methyl mercaptan (CH3SH), and dimethyl sulfide (CH3SCH3).

In the present study 46 (47.9%) of the participants were diagnosed with halitosis. This result is almost similar with a previously reported self-assessment of halitosis among Saudi patients, wherein 40% of the subjects were aware of the having halitosis [14,31]. The prevalence of halitosis in this study is higher than that reported by epidemiological studies in other communities that ranged between 2% and 40% [30,31,32,33]. The differences in the percentages of participants diagnosed with halitosis in these studies/populations could be related to the oral hygiene status, cultural, dietary, racial differences, and social habits [3].

According to the results of the current study the percentage of subjects diagnosed with halitosis in the group without fixed crowns was 32.69%, while in the group with fixed crowns was 65.90%. This significant difference indicated that the presence of fixed prostheses/crowns/restorations highly influences and contributes to the oral malodor or halitosis. The result of this study is in agreement with previous reports conducted by Zigurs et al. and Hossam, where fixed dental prostheses were found to intensify the development of halitosis; according to their conclusion it should be admitted that fixed prostheses make difficult or even completely impede the complex of oral cavity hygiene measures, thus intensifying the development of halitosis [19,32].

Another development of the current research was finding the association between the various fixed crown parameters and halitosis. The results showed high levels of VSCs in the participants with fixed crowns parameters, considered as faulty or defective parameters. The defective parameters investigated included subgingival margin, over-contoured margin, open-crown margin, over-contoured and under-contoured crowns. The recorded levels of the VSCs were significantly higher in the patients with these defective crown parameters as compared to the patients with nondefective crown parameters. This is an interesting finding considering the scarce availability of the research studies on the relationship between crown parameters and halitosis. In studies by Zigurs et al. [19] and Hossam [32], similar higher levels of halitosis associated with improper fixed dentures such as tooth crown laps, saddle intermediate parts, as well as fixed denture constructions that impede complex of mouth hygiene measures are reported. It has been reported that the prevalence of *Veillonella* spp. were higher in malodourous dentures, which is believed to be an important microorganism responsible for producing hydrogen sulfide in the oral cavity [33].

High rate of halitosis (47.91%) among both males (60.86%) and females (36%) subjects were observed in this current study, though the rate was lower among females. Studies have reported that females maintain better oral hygiene behavior, have a greater interest in oral health, and perceive their own oral health to be better than males [17].

Another study by Morita and Wang [34] examined the interaction between halitosis and demographic factors, oral hygiene, and periodontal status. Halitosis was found to be primarily associated with poor oral hygiene and gingival inflammation, with males having higher halitosis level than females [34]. The role of clinical periodontal variables in the progression of oral malodor is well researched and investigated [14,35]. The results of the current study are also no different, where the levels of plaque, bleeding index, PD, and CAL all showed higher levels in patients with halitosis. These findings are in line with several other research studies [12,35,36].

With regard to the study limitations, the results might have been affected by interparticipant variability in the oral health status and recording of their breath samples. Due to the multifactorial complexity of halitosis, each participant should be treated individually, rather than be categorized as a group. The evaluation of the various crown parameters in this study was performed by a trained clinician based on his experience. Further, longitudinal study design with exploring the prevalence of halitosis in crown types based on different materials is recommended. Additionally, further studies with larger sample size, tongue coating evaluation, and microbiological analysis is recommended. Constructions of artificial fixed crowns closely attached to the support tooth level and forms of intermediate parts, made with a hygienic angle, are recommended, in order to provide hygienic possibilities and reducing the risk of halitosis.

## 5. Conclusions

Within the limitations of this study, it could be concluded that:

- Presence of fixed dental crowns with defects significantly contributes to the oral halitosis.
- Improperly constructed crowns with defects significantly impair the hygienic conditions and microflora in oral cavity, resulting in the high prevalence of halitosis.
- The percentage of halitosis was higher among the male Saudi participants than females.

## Figures and Tables

**Table 1:** Demographic details of the participating patients in the study (*n* = 96).

Demographic Variables		Frequency	Percent	Valid Percent	Cumulative Percent
Gender	Male	46	47.9	47.9	47.9
	Female	50	52.1	52.1	100
Married	Yes	58	60.4	60.4	60.4
	No	38	39.6	39.6	100
Smoking	Never	60	62.5	62.5	62.5
	Former smoker	6	6.2	6.2	68.8
	Current	30	31.2	31.2	100
Dental visit	Every 6 months	22	22.9	22.9	22.9
	Every 12 months	24	25	25	47.9
	On pain	48	50	50	97.9
	Rarely	2	2.1	2.1	100
Brushing	Twice a week	16	16.7	16.7	16.7
	Once per day	23	24	24	40.6
	Twice or more per day	45	46.9	46.9	87.5
	Rarely	12	12.5	12.5	100
Education	Less than high school	1	1	1	1
	High school	18	18.8	18.8	19.8
	College	71	74	74	93.8
	High degree	6	6.2	6.2	100
Fixed restorations	Yes	52	54.2	54.2	54.2
	No	44	45.8	45.8	100
Total		96	100	100	

**Table 2:** Frequency table of different clinical and tested variables investigated in the study (*n* = 96).

Variables	Range	Frequency	Percent	Valid Percent	Cumulative Percent
Number of carious teeth	0	25	26.0	26.0	26.0
	1–5	49	51.0	51.0	63.3
	6–10	20	20.8	20.8	91.1
	11–13	2	2.0	2.0	100
Number of crowns	1	13	13.5	29.5	29.5
	2	17	17.7	38.6	68.2
	3	5	5.2	11.4	79.5
	4	9	9.4	20.5	100.0
H2S	<112	52	54.2	54.2	54.2
	≥112	44	45.8	45.8	100.0
CH3SH	<26	57	59.4	59.4	59.4
	≥26	39	40.6	40.6	100.0
CH3SCH3	≤8	60	62.5	62.5	62.5
	>8	36	37.5	37.5	100.0
Halitosis	No	50	52.1	52.1	52.1
	Yes	46	47.9	47.9	100.0
Total		96	100	100	

**Table 3:** Comparison of the concentration of volatile sulphur compound gases (VSCs) in participants with or without fixed crowns (*n* = 96).

Gases	Fixed Crowns	*n*	Mean	Std. Deviation	95% Confidence Interval for Mean		Minimum	Maximum	*p*-Value
					Lower Bound	Upper Bound			
H2S	No	52	87.00	83.18	63.84	110.16	10	382	0.000
	Yes	44	401.48	557.12	232.10	570.86	14	2263	
	Total	96	231.14	411.11	147.84	314.44	10	2263	
CH3SH	No	52	18.96	17.34	14.13	23.79	0	90	0.000
	Yes	44	72.61	89.68	45.35	99.88	0	334	
	Total	96	43.55	67.26	29.92	57.18	0	334	
CH3SCH3	No	52	6.85	9.64	4.16	9.53	0	66	0.075
	Yes	44	10.20	8.40	7.65	12.76	0	33	
	Total	96	8.39	9.20	6.52	10.25	0	66	

**Table 4:** Comparison of different crown parameters in relation to presence/absence of halitosis (*n* = 44).

Variables	Halitosis	Mean	Std. Deviation	95% Confidence Interval		Minimum	Maximum	*p*-Value
				Lower Bound	Upper Bound			
Supragingival Margin	No	65.27	25.08	51.38	79.16	25	100	0.000
	Yes	9.69	17.68	2.97	16.42	0.00	75	
Equigingival Margin	No	32.63	22.68	20.07	45.19	0.00	75	0.209
	Yes	25.21	15.62	19.27	31.15	0.00	50	
* Subgingival Margin	No	2.08	6.54	−1.53	5.70	0.00	25	0.000
	Yes	65.08	19.18	57.78	72.38	16.67	100	
* Over-Contoured Margin	No	2.91	7.03	−0.97	6.81	0.00	25	0.000
	Yes	59.62	19.67	52.14	67.10	25	93.75	
* Open-Crown Margin	No	4.30	8.46	−0.38	8.99	0.00	31.25	0.020
	Yes	13.43	13.28	8.37	18.48	0.00	50	
Optimal-Crown Margin	No	89.16	11.19	82.96	95.36	68.75	100	0.000
	Yes	26.07	20.89	18.12	34.02	0.00	62	
Ledge-Crown Margin	No	3.61	6.27	0.13	7.08	0.00	16.67	0.060
	Yes	0.86	3.22	−0.36	2.08	0.00	12.50	
* Over-Contoured Crown	No	5.00	19.36	−5.72	15.72	0.00	75	0.000
	Yes	48.13	30.85	36.39	59.86	0.00	100	
Normal-Contoured Crown	No	93.33	19.97	82.27	104.39	25	100	0.000
	Yes	47.27	28.22	36.53	58.00	0.00	100	
* Under-Contoured Crown	No	1.66	6.45	−1.90	5.24	0.00	25	0.395
	Yes	4.59	12.31	−0.08	9.28	0.00	50	

* Crown parameters considered defective clinically.

**Table 5:** Comparison of different periodontal variables in relation to presence/absence of halitosis (*n* = 96).

Variables	Halitosis	Mean	Std. Deviation	95% Confidence Interval		Minimum	Maximum	*p*-Value
				Lower Bound	Upper Bound			
PI	No	14.98	9.82	12.18	17.77	3.00	67.00	0.000
	Yes	69.32	19.42	63.55	75.09	10.00	98.00	
Bleeding index	No	11.55	6.45	9.72	13.38	1.10	36.00	0.000
	Yes	48.25	19.10	42.57	53.92	8.00	83.00	
PD	No	1.90	0.53	1.75	2.06	1.00	2.80	0.000
	Yes	4.31	1.33	3.91	4.70	1.80	7.10	
CAL	No	0.20	0.31	0.11	0.29	0.00	1.10	0.000
	Yes	3.10	1.36	2.69	3.50	0.00	6.28	

## Data Availability

The data presented in this study are available on reasonable request from the corresponding author. The data are not publicly available due to ethical requirements.

## References

[1] Wu J., Cannon R.D., Ji P., Farella M., Mei L. (2020). Halitosis: Prevalence, risk factors, sources, measurement and treatment–a review of the literature. Aust. Dent. J..

[2] Aydin M., Harvey-Woodworth C.N. (2014). Halitosis: A new definition and classification. Br. Dent. J..

[3] Silva M.F., Leite F.R., Ferreira L.B., Pola N.M., Scannapieco F.A., Demarco F.F., Nascimento G.G. (2018). Estimated prevalence of halitosis: A systematic review and meta-regression analysis. Clin. Oral. Investig..

[4] Liu X.N., Shinada K., Chen X.C., Zhang B.X., Yaegaki K., Kawaguchi Y. (2006). Oral malodor-related parameters in the Chinese general population. J. Clin. Periodontol..

[5] Renvert S., Noack M.J., Lequart C., Roldán S., Laine M.L. (2020). The Underestimated Problem of Intra-Oral Halitosis in Dental Practice: An Expert Consensus Review. Clin. Cosmet. Investig. Dent..

[6] Villa A., Zollanvari A., Alterovitz G., Cagetti M.G., Strohmenger L., Abati S. (2014). Prevalence of halitosis in children considering oral hygiene, gender and age. Int. J. Dent. Hyg..

[7] Yaegaki K., Coil J.M. (2000). Examination, classification, and treatment of halitosis; clinical perspectives. J. Can. Dent. Assoc..

[8] Kapoor U., Sharma G., Juneja M., Nagpal A. (2016). Halitosis: Current concepts on etiology, diagnosis and management. Eur. J. Dent..

[9] Rösing C.K., Loesche W. (2011). Halitosis: An overview of epidemiology, etiology and clinical management. Braz. Oral Res..

[10] Lauritano D., Boccalari E., Di Stasio D., Della Vella F., Carinci F., Lucchese A., Petruzzi M. (2019). Prevalence of oral lesions and correlation with intestinal symptoms of inflammatory bowel disease: A systematic review. Diagnostics.

[11] Guedes C.C., Bussadori S.K., Weber R., Motta L.J., da Mota A.C.C., Amancio O.M.S. (2019). Halitosis: Prevalence and association with oral etiological factors in children and adolescents. J. Breath Res..

[12] Alzoman H. (2008). Periodontal disease and halitosis. Saudi Dent. J..

[13] Ye W., Zhang Y., He M., Zhu C., Feng X.-P. (2019). Relationship of tongue coating microbiome on volatile sulfur compounds in healthy and halitosis adults. J. Breath Res..

[14] Alzoman H. (2021). The association between periodontal diseases and halitosis among Saudi patients. Saudi Dent. J..

[15] Tangerman A. (2002). Halitosis in medicine: A review. Int. Dent. J..

[16] Hampelska K., Jaworska M.M., Babalska Z.Ł., Karpiński T.M. (2020). The role of oral microbiota in intra-oral halitosis. J. Clin. Med..

[17] Scully C., El-Maaytah M., Porter S.R., Greenman J. (1997). Breath odor: Etiopathogenesis, assessment and management. Eur. J. Oral Sci..

[18] Tangerman A., Winkel E.G. (2013). Volatile Sulfur Compounds as The Cause of Bad Breath: A Review. Phosphorus. Sulfur. Silicon. Relat. Elem..

[19] Zigurs G., Vidzis A., Brinkmane A. (2005). Halitosis manifestation and prevention means for patients with fixed teeth dentures. Stomatologija.

[20] Costacurta M., Petrini M., Biferi V., Arcuri C., Spoto G., Brescia A., Docimo R. (2020). Dental prosthesis and halitosis: Evaluation of oral malodor in patients with and without a dental prosthesis. J. Osseointegration.

[21] Sinjari B., Murmura G., Caputi S., Ricci L., Varvara G., Scarano A. (2013). Use of Oral Chroma in the assessment of volatile sulfur compounds in patients with fixed protheses. Int. J. Immunopathol. Pharmacol..

[22] Mbodj E., Faye B., Faye D., Seck M., Sarr M., Ndiaye C., Dabo P., Diallo P. (2011). Prevalence of halitosis in patients with dental prostheses in Senegal. Med. Tropic. Rev. Corps Sante Colon..

[23] Huang J., Li C.-Y., Jiang J.-H. (2018). Effects of fixed orthodontic brackets on oral malodor: A systematic review and meta-analysis according to the preferred reporting items for systematic reviews and meta-analyses guidelines. Medicine (Baltimore).

[24] Kaygisiz E., Uzuner F.D., Yuksel S., Taner L., Çulhaoğlu R., Sezgin Y., Ateş C. (2015). Effects of self-ligating and conventional brackets on halitosis and periodontal conditions. Angle Orthod..

[25] Swelem A.A., Gurevich K.G., Fabrikant E.G., Hassan M.H., Aqou S. (2014). Oral Health–Related Quality of Life in Partially Edentulous Patients Treated with Removable, Fixed, Fixed-Removable, and Implant-Supported Prostheses. Int. J. Prosthodont..

[26] Ali Z., Baker S.R., Shahrbaf S., Martin N., Vettore M.V. (2019). Oral health-related quality of life after prosthodontic treatment for patients with partial edentulism: A systematic review and meta-analysis. J. Prosthet. Dent..

[27] Tan K., Pjetursson B.E., Lang N.P., Chan E.S. (2004). A systematic review of the survival and complication rates of fixed partial dentures (FPDs) after an observation period of at least 5 years: III. Conventional FPDs. Clin. Oral Implants Res..

[28] Alnazzawi A. (2018). Effect of fixed metallic oral appliances on oral health. J. Int. Soc. Prev. Commun. Dent..

[29] Van den Velde S., van Steenberghe D., Van hee P., Quirynen M. (2009). Detection of Odorous Compounds in Breath. J. Dent. Res..

[30] Tangerman A., Winkel E.G. (2008). The portable gas chromatograph OralChroma™: A method of choice to detect oral and extra-oral halitosis. J. Breath Res..

[31] Almas K., Al-Hawish A., Al-Khamis W. (2003). Oral Hygiene Practices, Smoking Habits, and Self-Perceived Oral Malodor Among Dental Students. J. Contemp. Dent. Pract..

[32] Eid H. (2012). Effects of different fixed partial dentures and different margin positions on the halitosis level. Alazhar. Dent. J..

[33] Yitzhaki S., Reshef L., Gophna U., Rosenberg M., Sterer N. (2018). Microbiome associated with denture malodour. J. Breath Res..

[34] Morita M., Wang H.-L. (2001). Association between oral malodor and adult periodontitis: A review. J. Clin. Periodontol..

[35] Silva M.F., Cademartori M.G., Leite F.R., Lopez R., Demarco F.F., Nascimento G.G. (2017). Is periodontitis associated with halitosis? A systematic review and meta-regression analysis. J. Clin. Periodontol..

[36] De Geest S., Laleman I., Teughels W., Dekeyser C., Quirynen M. (2016). Periodontal diseases as a source of halitosis: A review of the evidence and treatment approaches for dentists and dental hygienists. Periodontology 2000.

